# Characterization of the *Conus bullatus *genome and its venom-duct transcriptome

**DOI:** 10.1186/1471-2164-12-60

**Published:** 2011-01-25

**Authors:** Hao Hu, Pradip K Bandyopadhyay, Baldomero M Olivera, Mark Yandell

**Affiliations:** 1 Eccles institute of Human Genetics, University of Utah, and School of Medicine, Salt Lake City, UT 84112, USA; 2 Department of Biology, University of Utah, Salt Lake City, UT 84112, USA

## Abstract

**Background:**

The venomous marine gastropods, cone snails (genus *Conus*), inject prey with a lethal cocktail of conopeptides, small cysteine-rich peptides, each with a high affinity for its molecular target, generally an ion channel, receptor or transporter. Over the last decade, conopeptides have proven indispensable reagents for the study of vertebrate neurotransmission. *Conus bullatus *belongs to a clade of *Conus *species called *Textilia*, whose pharmacology is still poorly characterized. Thus the genomics analyses presented here provide the first step toward a better understanding the enigmatic *Textilia *clade.

**Results:**

We have carried out a sequencing survey of the *Conus bullatus *genome and venom-duct transcriptome. We find that conopeptides are highly expressed within the venom-duct, and describe an *in silico *pipeline for their discovery and characterization using RNA-seq data. We have also carried out low-coverage shotgun sequencing of the genome, and have used these data to determine its size, genome-wide base composition, simple repeat, and mobile element densities.

**Conclusions:**

Our results provide the first global view of venom-duct transcription in any cone snail. A notable feature of *Conus bullatus *venoms is the breadth of A-superfamily peptides expressed in the venom duct, which are unprecedented in their structural diversity. We also find SNP rates within conopeptides are higher compared to the remainder of *C. bullatus *transcriptome, consistent with the hypothesis that conopeptides are under diversifying selection.

## Background

Next-generation sequencing techniques have opened up new opportunities for genomics studies of new model organisms [1]. Many of these organisms are not amenable to classical genetic techniques; thus their sequenced and annotated genomes are the central resource for experimental studies. The popularity of the Planarian *Schmidtea mediterranea*, which can regenerate complete animals from fragments of its body, with stem-cell researchers is one example [2]. The Cone snail is another.

The cone snails (genus *Conus) *belong to the superfamily Conoidea which probably includes over 10,000 venomous gastropods [3]. The venom from each of the species of cone snails includes a mixture of small cysteine-rich peptides, which are used to immobilize their prey. These small peptides (~15 to 40 amino acids in length) have exquisite specificity for different isoforms of ion channels, receptors and transporters [4]. Their disulfide scaffold restricts the conformational space available to a peptide. However, the combination of variable intervening amino acids and their posttranslational modifications enable a spectrum of specific interactions with their target molecules. A typical conopeptide precursor is comprised of three regions: an N-terminal signal peptide, a pro-region, and a mature peptide region. The N-terminal sequence is usually much more conserved than the mature peptide, possibly due to the diversifying selection on the latter [5]. Conopeptides are classified into super-families, mainly based on the conserved signal peptide and different cysteine patterns observed within the mature peptide.

Conopeptides serve as specific neurobiological tools for addressing specific receptors and channels, and are also valuable lead compounds for therapeutic evaluation. A conopeptide, ω-MVIIA (commercially known as Prialt, ziconotide) isolated from *Conus magus*, has been approved by FDA for the treatment of chronic pain [6,7]. In addition, other conopeptides are also being evaluated for the treatment of pain and epilepsy [8-11]. It is estimated that the venom of a single species of *Conus *may contain as many as 200 different venom peptides [4,12]. This raises the possibility that the 500-700 species of cone snails may provide upwards of 100,000 compounds of potential pharmacological interest, perhaps more when all the members of superfamily *Conoidea *are considered.

We have carried out a sequencing survey of the *Conus bullatus *genome and venom-duct transcriptome. *Conus bullatus *is a fish-hunting cone snail that together with *C. cervus *and *C. dusaveli *are members of the subgenus *Textilia *(Swainson, 1840). This is probably the least understood group of fish-hunting *Conus*. All are from the Indo-Pacific region (Pacific and Indian oceans from Hawaii through South Africa). *Conus bullatus *is the only accessible member of this clade of species; all others are rare and from deep water. *C. bullatus *is found from the intertidal zone to about 240 m, most commonly from slightly subtidal to 50 m, *C. cervus *between 180-400 m and *C. dusaveli *50-288 m [13].

**Figure 1:**
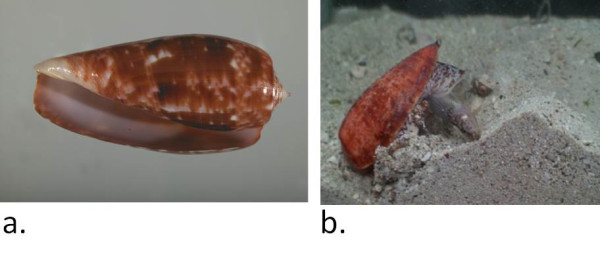
**Conus bullatus and its feeding preference.** a. Shell of *Conus bullatus*; b. Prey capture by *Conus bullatus*.

The pharmacology of the *Textilia *is thus still poorly characterized, and the genomics analyses presented here provide the first step toward a better understanding the enigmatic *Textilia *clade. The biology of the *Conus *species that belong to the Textilia clade is mostly unknown, but we recently documented the prey capture behavior of *Conus bullatus *(Figure 1). The general strategy appears to be analogous to that first established for *Conus purpurascens *[14], with one group of venom peptides causing a rapid tetanic immobilization, and a second set eliciting a block of neuromuscular transmission. Multiple venom peptides that act coordinately to achieve a particular physiological endpoint are referred to as "conopeptides cabals" [15]. The fish-hunting cone snails generally have both a "lightning-strike cabal" and a "motor cabal" leading to the tetanic immobilization and neuromuscular block, respectively. A video of *Conus bullatus *has documented the most rapid tetanic immobilization of prey observed for any fish-hunting cone snail. (http://www.hhmi.org/biointeractive/biodiversity/2009_conus_bullatus.html).

Venom studies in *Conus bullatus *have already yielded results of exceptional pharmacological interest. The best characterized *bullatus *venom component, alpha-conotoxin BuIA is a small peptide antagonist of nicotinic receptors that has become the standard pharmacological tool for differentiating between nicotinic receptors that carry two closely related subunits, β2 and β4. These receptors are of considerable interest in Parkinson's disease [16]. More recently, the μ-conotoxins, peptides with 3 disulfide bonds that are antagonists of voltage-gated Na channels have also been characterized from *Conus bullatus *[17]. These peptides appear to have novel subtype selectivity for the different molecular isoforms of voltage-gated Na channels [17]. Thus, they provide a promising neuropharmacological lead to developing an entirely new pathway to differentiate between different voltage-gated Na channel subtypes. Clearly, better cone snail genomics resources would aid these studies; however, few such resources exist as yet for *Conus *studies, and none for *C. bullatus*.

The cone snails are being extensively investigated as a source of peptidic pharmacological agents (ligands) with exquisite specificity for different subtypes of receptors in the central nervous system. In keeping with this main goal it is not surprising that most of the available nucleic acid sequences from *Conus *are a catalogue of these compounds present in the venom. In addition, partial sequences of a few mitochondrial (ribosomal RNA and COI) and nuclear genes [18-23] have also been determined to ascertain the phylogenetic relationship among cone snails.

Previous work has used traditional molecular biology approaches to clone genes encoding members of specific conopeptide super-families [20,24-26], and EST sequencing in another *Conus *snail has identified conopeptides [27,28]. However, to date, no high-throughput sequencing approach on the whole mRNA reservoir of a *Conus *venom-duct has been attempted.

We have used RNA-seq [29] to identify and profile the expression of conopeptides and post-translational modification enzymes implicated in venom production. Our results provide the first global view of venom-duct transcription. Our shotgun genomic survey complements our RNA-seq data, and is also the first reported for a cone snail. Knowledge of several marine gastropod genomes will provide a first step toward the molecular understanding of numerous traits unique to these species. Accordingly, we have used these data to determine the suitability of the genome for sequencing and assembly with 2^nd ^generation technologies, determining genome-wide base composition, sequence heterozygosity, simple repeat, and mobile element densities within the *C. bullatus *genome.

As we show, our RNA-seq and genomic datasets can be combined to enable analyses not possible with either dataset alone. For example, the transcriptome assembly has allowed us to explicitly test the hypothesis that conopeptides are under diversifying selection [5]. We have also developed a novel method for estimating genome size using RNA-seq and genomic shotgun sequences, which we present here. The approach is accurate, and should prove useful for any researcher seeking to determine the size of an emerging model organism [1] genome using 2^nd ^generation sequencing data.

## Results

### Sequence datasets

We generated 96,379,716 Illumina paired 59-mers and 55,699,572 paired 60-mers for the genome. The average insert size of the paired-end library is 200nt. We also isolated venom-duct poly-A mRNA and sequenced it using both Illumina and Roche technologies. On the Illumina platform, we generated 102,278,116 paired 79-mers with a median insert size of 340bp. The Roche 454 platform generated 848,394 reads with average read length of 248bp. Many cDNA reads from the Illumina platform have low-quality 3' ends, which could be due to either to the small amounts of mRNA used in our experiments, or instrument error during sequencing or processing. We removed 3'end sequences from the reads with phred quality values of 2.

### Genome-wide GC content

We randomly selected 30 million genomic reads using the process described in the Methods section (see section Simulated Read Sets) and determined their GC content. This procedure gives an estimated GC content for the *C. bullatus genome *of 42.88%. To validate this method, we also simulated 1 million randomly sampled 60-mers from the *D. melanogaster *genome and performed the same experiment, which gives 41.87%, an estimate in good agreement with the actual GC content (41.74%.) of the *D. melanogaster *genome.

### Genome-wide Repeat Content

**Figure 2:**
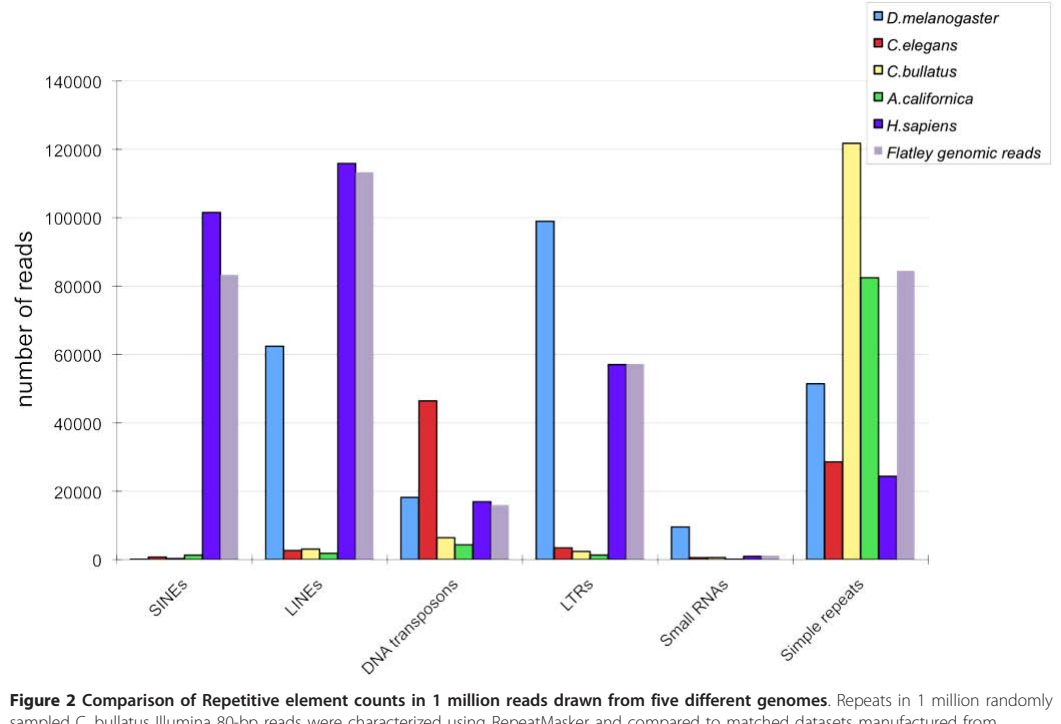
**Comparison of Repetitive element counts in 1 million reads drawn from five different genomes**. Repeats in 1 million randomly sampled C. bullatus Illumina 80-bp reads were characterized using RepeatMasker and compared to matched datasets manufactured from simulated reads from three other sequenced genomes and real reads from Flatley genome. X-axis: repeat-class. Y-axis: counts.

We took three approaches to characterize the repeat content of the *C. bullatus *genome. First, we ran RepeatMasker on 1 million randomly selected *C. bullatus *genomic reads, comparing the results to a matched human, *Caenorhabditis elegans*, *Drosophila melanogaster*, and *Aplysia californica *(a mollusk) datasets of simulated reads, as well as real human genome reads[30] (see Methods for details); these datasets match the *Conus *data precisely as regards number of reads, distance between pairs, read lengths, and (among the simulated sets) base quality (see Methods for details). Comparisons of the simulated human reads to real human reads (purple and grey columns in Figure 2), indicates that the simulated human reads closely match the real reads as regards repeat content for all repeat classes except simple repeats. We speculate that this is because many simple repeats (e.g., those near telomeres and centromeres) are designated as "N" in the reference human genome; hence, a random sampling of segments of the human reference assembly under represents its simple repeat content.

RepeatMasker [31] and RepBase [32] lack extensive libraries of repeats for mollusks, which will compromise the ability of RepeatMasker to identify interspersed repeats in the two mollusk datasets. Although, this fact does not complicate direct comparison of *C. bullatus *and *Aplysia californica*, with regards to the relative numbers of conserved interspersed repeats, it does complicate absolute measurements and comparisons to the other genomes (Figure 2). The ability of RepeatMasker to identify simple repeats, however, is less impacted by the lack of well-characterized repeat libraries for mollusks. This fact together with comparison with J. Flatley's genomic reads [30](Figure 2) suggests that *C. bullatus *is significantly enriched for simple repeats relative to the other invertebrates, and slightly so (1.44 fold) compared to human.

We also used RECON [33] to identify novel, high-copy genomic sequences that may be interspersed repeats in the *C. bullatus *genome. For this analysis we used our *C. bullatus de novo *genomic assembly (see Methods). In total, we found 115 genomic contigs present in 10 or more copies, with an average length of 544bp. Among these genomic sequences, 5 are homologous with known LINE members that were not detected by RepeatMasker in first repeat analysis. Of the remaining contigs, 9 have significant homology with G-protein receptors; 2 have significant homology with lipoprotein receptors; 1 has a leucine-rich repeat structure. These are probably high-copy number genomic regions but are not interspersed repeats. The remaining contigs have little homology with known interspersed repeats, however, a significant fraction of them have either strong homology to nuclease proteins or weak homology with rRNA and tRNA genes-both common motifs in LINE elements. Running RepClass [33] over these 115 genomic contigs confirmed that 20 contigs have LINE-like structures or are significantly homologous to known LINEs. Including this set would increase the percentage of the *C. bullatus *genome with LINE homology from 0.24% to 0.56%.

**Figure 3:**
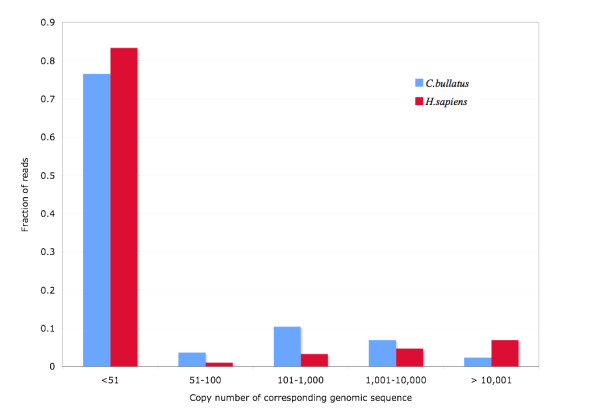
**Profile of proportion of the genomic sequences with each copy number**. Generated from all-by-all blast analysis of one million *C. bullatus *and *H.sapiens *reads each against themselves. The number of read partners is converted to copy-number of corresponding genomic sequence. X-axis: each bin's label gives the minimum and maximum copy numbers in the genome. Y-axis: fraction of reads falling into that bin.

Because novel forms of retro-transposons might not have been identified in our RepeatMasker experiment, or some unknown bias in the ABySS [34] assembler might have caused us to underestimate the numbers of novel repeats identified with RECON, we devised a third experiment, that controls for both of these possibilities. In this experiment, we took the same read-datasets used in our RepeatMasker analysis (Figure 2), and performed an all-against-all BLAST [35] search of the *C. bullatus *reads against themselves, and repeated the same experiment for a matched set of simulated reads from *H. sapiens *(see Methods for details). For reasons of computational complexity we choose to limit this analysis to only one target genome: *H. sapiens*, because it is the most repeat rich of any in our dataset and its genome is nearly the same size as the *C. bullatus *genome. We then tallied the percentage of reads having one BLAST hit, two hits and so on. For each read, its number of hits can be used to obtain an estimate of the copy-number of its sequence within the genome (see Methods). This allows us to estimate the proportion of high-copy number genomic sequences within the *Conus *genome and to make comparisons to the human genome (Figure 3). This experiment presumes no prior knowledge of the repeat content of the genome. We also used the 'SEG' option with WU-BLAST [36] to exclude hits between reads consisting only of low complexity and/or simple sequence repeats. By using BLAST with the SEG option any reads consisting entirely of low complexity or simple sequence repeats will have no hits.

This analysis reveals much about the repeat content of *Conus *compared to that of the Human genome. First, the *Conus *genome has a larger proportion of high copy-number sequences (presumably interspersed repeats) compared to human. This is shown by the fact that 23% of *Conus *reads (compared to 16% in human) have numbers greater than 50. By looking into this group of human reads, we confirmed that 91% of these are homologous to known interspersed repeats. Second, the human dataset (and hence the human genome as compared to the *Conus *genome) has 3× as many genomic sequences with a copy number above 10,000 compared to *Conus *(6.9% versus 2.4%). These sequences are mostly non-LTR elements that exists in extremely high copy number; running Repeatmasker over these human genome reads showed that 75% of these genomic regions are SINEs and another 20% are LINEs, supporting this hypothesis. Taken together, our results show that although the *Conus *genome is enriched for interspersed repeats compared to human, it has far fewer non-LTR repetitive elements.

### A partial genome assembly

A previous estimate based upon cytology, placed the *Conus bullatus *genome at around 3 billion base pairs [37]. If true, our 60 bp paired-end Illumina dataset would provide 3× coverage. Although this is insufficient to produce anything near a complete genome assembly, a partial genome assembly is still desirable for some analyses. We used ABySS to produce a partial assembly 201 million base pairs in length with an N50 value of 182 bp (See Methods for details). This accounts for ~7% of the total length of the *C. bullatus *genome. To estimate the quality of our genome assembly, we simulated 8.7 million 60bp-long Illumina reads from the *D. melanogaster *genome (3× coverage), with the same base-calling accuracy distribution as in our *Conus *genomic reads. To do so we used the procedure described in the Methods section. This process gives 3× coverage over the *Drosophila *genome with the same error rates as our *C. bullatus *reads. Assembling these reads with ABySS with the same parameters produced a *Drosophila *assembly with an N50 of 143 bp and total sequence length of 16 MB, which accounts for roughly 10% of the fly genome. Thus the two assemblies are of comparable quality.

### Assembly of the venom-duct transcriptome

We assembled our Illumina RNA-seq reads from the *C. bullatus *venom-duct with ABYSS (see methods for details). This produced 525,537 contigs of 60bp or greater in length and having a total length of 57 MB. We chose 60bp as minimum contig size because conopeptides can be as short as 20 amino acids. The 454 reads were generated and assembled by Roche.

### Annotation of transcriptome

To determine the percentage of the total *C. bullatus *proteome sampled one or more times in our Illumina and Roche transcriptome datasets, we took the core eukaryotic protein set from CEGMA [38], which is comprised of 248 core proteins that generally lack paralogs in the eukaryotes [38,39], and asked what percentage of these proteins are found in the combined Illumina or Roche assemblies. Using BLASTX, 211 out of 248 proteins (85%) are found (E < = 1e^-7^).

To annotate the transcriptome assembly we ran WU-BLASTX on the ABySS Illumina assembly against UniProtKB database [40]. 7,691 unique UniProtKB proteins have significant homology with one or more transcriptome contigs. We also mapped those contigs no shorter than 200bp to GO [41] terms for biological process, molecular function and cellular component. As a control, we applied the same approach to the annotated *C. elegans, D. melanogaster *and *H. sapiens *transcriptomes and compared the proportion of genes assigned to each GO term in these organisms to our transcriptome assembly results (Additional File 1). Note that is not a comparison of expression levels, but rather a comparison using GO of which genes were represented in our transcriptome assembly. In other words, the *relative *proportions of all GO gene categories associated with our *C. bullatus *contigs was found to be similar to the relative proportions of genes assigned to the same GO categories for *C. elegans, D. melanogaster *and *H. sapiens *transcriptomes. We found that the resulting GO profiles are highly similar for all four organisms. This finding, together with our observation that 85% of CEGMA proteins are represented in the assembly, suggests that we have sampled a wide swath of the *C. bullatus *transcriptome.

### Identification of Conopeptides in RNA-seq data

We searched our combined Illumina and Roche transcriptome assemblies for significant homology to a set of known conopeptides collected from ConoServer [42], using the procedure described in the Methods section. We find that, as might be expected, conopeptides are transcribed at high levels in the venom duct; the depth of coverage of the putative conopeptides is 102× versus 33× for the remainder of the transcriptome.

Whenever possible, we assigned each of our putative conopeptide contigs to a conopeptide superfamily, by significant homology to signal sequences that are characteristic of each superfamily (see Methods for details). In total, we were able to assign 543 contigs a unique conopeptide super-family. We find that, as in most *Conus *species examined so far, the O1, M, A and T superfamilies were represented by the greatest number of distinct contigs. We also observed that mRNA abundance levels followed this same general pattern with respect to superfamilies (Table 1). Besides these well represented superfamilies, we also found small number of conopeptides belonging to the rarer in I2 and J conopeptide super-families in *Conus bullatus*, which account for ~0.4% of total putative-conopeptide transcripts.

**Table 1:** Superfamilies of *C. bullatus *conopeptides identified by RNA-seq.

Conopeptide Super Family	*C. bullatus *RNA-seq data	Conoserver reference sequences
**T**	15%	13%
**A**	17%	19%
**M**	20%	9%
**O2**	4%	5%
**O1**	44%	40%
**Other**	< 1%	14%

Percentages for *C. bullatus *refer to percentage of venom-duct RNA-seq reads belonging to a given superfamily. Globally the distribution parallels that for reference conopeptide sequences by class available on Conserver, although rare classes are under-sampled.

In total, we identified 2,410 putative conopeptide contigs. Most of these contigs are short (with the N50 of 69bp), and do not contain the full-length sequence of the conopeptide precursor. Nevertheless, we were able to identify a few complete conopeptides (mainly from the Roche data), and a selection of 30 putative complete and partial conopeptide sequences are presented in (Table 2). The conopeptides listed belong to the O, M, A, J, contryphan and conkunitzin super-families with O- being the most abundant. While conopeptides belonging to the I2, T, con-ikot-ikot, and conantokin super-families could be identified in the Blast analysis; the contig lengths and frameshifts associated these hits precluded the generation of a high confidence protein sequence.

**Table 2:** Translated transcripts containing putative toxin sequences.

**O-superfamily: C-C-CC-C-C**		
	**1**.	MKLTCVAIVAVLLLTACQLITAEDSRGTQLHRALRKTTKLSVSTR***C****KGPGAK****C****LKTMYD****CC****KYS****C****SRGR****C***
	**2**.	MKLTCVLIIAVLFLTAITADDSRDKQVYRAVGLIDKMRRIR*ASEG****C****RKKGDR****C****GTHL****CC****PGLR****C****GSGRAGGA****C****RPPYN*
	**3**.	MKLMCVLIVSVLVLTACQLSTADDTRDKQKDRLVRLFRKKRDSSDSGLLPR*T****C****VMFGSM****C****DKEEHSI****CC****YE****C****DYKKGI****C****V*
	**4**.	MKLTCVVIVAVLLLTACQLIIAEDSRGTQLHRALRKATKLSVSTR*T****C****VMFGSM****C****DKEEHSI****CC****YE****C****DYKKGI****C****V*
	**5**.	MKLTCVLIVAVLFLTACQLATAENSREEQGYSAVRSSDQIQDSDLKLTK*S****C****TDDFEP****C****EAGFEN****CC****SKS****C****FEFEDVYV*C*GVSIDYYDSR
	**6**.	MKLICVFIVAVLLLTACQLNAADDSRDTQKHRALRSTTKLSMSKK*DS****C****VPDGDS****C****LFSRIP****CC****GT****C****SSRSKS****C***V*G
	**7**.	MKLTCMMIVTVLFLTAWTFVTADDSTYGLKNLLPKARHEMMNPEAPKLNKK*DE****C****SAPGAF****C****LIRPGL****CC****SEF****C****FFA****C****F *[67]
	**8**.	AEDSRGTQLHRALRKATKLSESTR***C****KRKGSS****C****RRTSYD****CC****TGS****C****RNGK*C*G
	**9**.	AVLLLTACQLITAEDSRDTQKHRALRSDTKLSMLTLR***C****ATYGKP****C****GIQND****CC****NI****C****DPARRT****C****T*
	**10**.	DSRGTQLHRALRKATILSVSAR***C****KLSGYR****C****KRPKQ****CC****NLS****C****GNYM*C*G
	**11**.	ACQLITAEDSRGTQLHRALRSTSKVSK*STS****C****VEAGSY****C****RPNVKL****CC****GF****C****SPYSKI****C****MNFPKN*
	**12**.	TAEDSRGTQLHRALRKATKLPVSTR***C****ITPGTR****C****KVPSQ****CC****RGP****C****KNGR****C****TPSPSEW*
	**13**.	AEDSRGTQLHRALRKTTKLSLSIR***C****KGPGAS****C****IRIAYN****CC****KYS****C****RNGK****C****S*
	**14**.	AACQLGTAASFARDKQDYPAVRSDGRQDSKDSTLDRIAKR***C****SEGGDF****C****SKNSE****CC****DKK****C****QDEGEGRGV****C****LIVPQNVILLH*
**M-superfamily: CC-C-C-CC**		
	**15**.	MLKMGVLLFTFLVLFPLATLQLDADQPVERYADNKQDLNPDER*MIFLFGG****CC****RMSS****C****QPPPV****C****N****CC****AKQDLNPDER*
	**16**.	DQPADRPAERMQDDISSEQNPLLEKR*VGER****CC****KNGKRG****C****GRW****C****RDHSR*CC*GRR [17]
	**17**.	*GLY****CC****QPKPNGQMM****C****NRW****C****EINSR*CC*GRR
**A-superfamily: CC-C-C; CC-C-C-C-C**		
	**18**.	MGMRMMFTVFLLIVLATTVVSFSTDDESDGSNEEPSADQTARSSMNR*APG****CC****NNPA****C****VKHR*C*G [68]
	**19**.	MGMRMVFTVFLLVVLATTVVSFTSDRASDGRNAAANDKASDLAALAVR*G****CC****HDIF****C****KHNNPDI*C*G
	**20**.	MGMRMRMMFTVFLLVVLANTVVSFPSDRDSDGADAEASDEPVEFER*DENG****CC****WNPS****C****PRPR****C***T*GRR [68]
	**21**.	DGANAEATDNKPGVFER*DEKK****CC****WNRA****C****TRLVP****C****SK*
	**22**.	SDRASDGRNAAANDRASDLVALTVR*G****CC****TYPP****C****AVLSPL****C****D*
	**23**.	MGMRMMVTVFLLGVLATTVVSLRSNRASDGRRGIVNKLNDLVPQYWTE***CC****GRIGPH****C****SR****C****I****C****PEVV****C***PKN*G
	**24**.	MGMRMMVTVFLLVVLATTVVSLRSNRASDGRRGIVNKLNDLVPKYWTE***CC****GRIGPH****C****SR****C****I****C****PEVA****C***PKN*G
	**25**.	MGMRMMVTVFPLVVLATTVVSLRSNRASDGRRGIVNKLNDLVPKYWTE***CC****GRIGPH****C****SR****C****I****C****PGVV****C***PKR*G
	**26**.	LVVLATTVVSFRSNRASDGRKIAVNKRRR*ELVVPPGKLRE****CC****GRVGPM****C****PK****C****M****C****PPRR****C***
	**27**.	ASDGRNAVVHER*APELVVTATTT****CC****GYDPMTI****C****PP****C****M****C****THS****C***PPKRKP*GRRND
**J-superfamily**		
	**28**.	MTSVQSATCCCLLWLVLCVQLVTPDSPATAQLSRHLTAR*VPVGPALAYA****C****SVM****C****AKGYDTVV****C****T****C***TRRRG*VVSSSI
**Contryphan**		
	**29**.	MGKLTILVLVAAVLLSTQVMGQGDRDQPAARNAVPRDDNPGGASAKLMNLLHRSKCPWSPWC*G
**Conkunitzin**		
	**30**.	MEGRRFAAVLILPICMLAPGAVASKR*WTRPSV****C****NLPAESGTGTQSLKRFYYNSDKMQ****C****RTFIYKGNGGNDNNFPRTYD****C****QKK****C***LYRP*G

Cysteine motifs are shown next to the superfamilies. The underlined residues indicate presumed propeptide cleavage site ascertained by analogy to previously isolated toxins; * indicate probable amidation at the C-terminal residue after cleavage of the following G residue. In the case of 23,24,25,26 where the propeptide cleavage site is uncertain, we have indicated the cleavage site at the basic residues (K) proximal to the presumed toxin sequence. The peptides Bu 7, 16, 18 and 20 have been previously characterized.

A notable feature of the *Conus bullatus *transcriptome analysis is the breadth of A-superfamily peptides expressed in the venom duct, which are unprecedented in their structural diversity (Table 3). In most Conus species, the predominant structural classes of A-peptides is the α4/7 subfamily; in fish-hunting cone snails, additional subclasses are the α3/5 subfamily and κA conotoxins (in species of the *Pionoconus *clade) and the αA conotoxins (in species of the *Chelyconus *clade). The *Conus bullatus *transcriptome includes an mRNA encoding a κA conotoxin (Bu27), which is unambiguous in its identity. There is also a single member of the α4/7 subfamily (Bu19) of unknown function, which is strikingly different in sequence from all other Conus venom peptides in this group. Although no member of the αA family or the α3/5 subfamilies were found, 8 other A superfamily peptides were identified. Together these comprise a greater range of structural diversity in the A-superfamily than has been found in any other venom. Three subclasses of α-conotoxins represented two different α4/4 peptides (Bu18 and 20), one α4/5 peptide (Bu21) and one α4/6 peptide (Bu22). Unique to *C. bullatus *are the four A peptides with 3 disulfide bonds (Bu 23, 24, 25 and 26) which are divergent from both κA and αA families. It is notable that although these comprise a significant fraction of the total complement of A-superfamily peptides in *C. bullatus*, similar peptides have not been reported from any other species thus far. Thus, it appears that *Conus bullatus*, and potentially the *Textilia *clade of Conus species, has explored novel evolutionary pathways in generating their complement of A-gene superfamily peptides.

**Table 3:** Sequence diversity and classification of A-superfamily conopeptides from *Conus bullatus*

Subclasses of A-superfamily peptides (Mature toxin sequences)		
		
α4/4		
	Bu18	APG**CC**NNPA**C**VKHRC*
	Bu20	DENG**CC**WNPS**C**PRPR**C**T*
		
α4/5		
	Bu21	**CC**WNRA**C**TRLVP**C**SK
		
α4/6		
	Bu22	G**CC**TYPP**C**AVLSPL**C**D
		
α4/7		
	Bu19	G**CC**HDIF**C**KHNNPDIC*
		
κA		
	Bu27	APELVVTATTT**CC**GYDPMTI**C**PP**C**M**C**THS**C**PPKRKP*
		
κA-like		
	Bu23	**LNDLVPQYWTECC**GRIGPH**C**SR**C**I**C**PEVV**C**PKN*
	Bu24	YWTE**CC**GRIGPH**C**SR**C**I**C**PEVA**C**PKN*
	Bu25	YWTE**CC**GRIGPH**C**SR**C**I**C**PGVV**C**PKR*
		
	Bu26	LRE**CC**GRVGPM**C**PK**C**M**C**PPRR**C**

*C-terminal is amidated. We have assumed that the proteolytic cleavage site is at the basic residue proximal to the presumed toxin sequence.

### SNP rates in conopeptides

We also compared the single nucleotide heterozygosity level within the transcripts encoding conopeptides to the rest of the transcriptome. To reduce false negative rates, we restricted our analysis to transcriptome contigs having coverage depths of 10× or more. Our rationale being that SNPs within low-coverage contigs might be missed, leading us to underestimate the actual SNP rate. For the transcriptome as a whole, the SNP rate is 0.0035 (102,955 SNPs in 29.5 MB of high-coverage contigs). By contrast, the single nucleotide polymorphism rate within conotoxin contigs is 0.011 (1146 SNPs in 105,259bp of high-coverage conotoxin contigs; this is 64% of all conotoxin contigs by length). The 3.1-fold higher SNP rate within conopeptides contigs is consistent with the hypothesis that conopeptides are under diversifying selection.

### Candidate post-translational processing enzymes

Conopeptides contain post-translationally modified amino acids. These modifications play an important role in conferring target specificity. The most ubiquitous modification is the formation of disulfides leading to proper conotoxin folding; this mediated by disulfide isomerases, chaperones and enzymes involved in redox biochemistry. From an examination of transcriptome sequences we have identified partial and complete sequences of several chaperones and thiol-disulfide oxidoreductases that are likely to be involved in the redox biochemistry of conotoxin folding (Additional File 2).

We identified some of the enzymes that are presumed to catalyze correct disulfide connectivity within conopeptides [43-46]. These include members of the QSOX family of sulfhydryl oxidases, Ero oxidases and protein disulfide isomerases (PDIs). PDIs also have chaperone-like activity and prevent protein aggregation. We have identified three isoforms of protein disulfide isomerase (PDI) and four members belonging to different subfamilies of PDIs. Two of these are members of the P5 subfamily. We also identified a transcript related to human PDIRs, which carry out oxidation-isomerization functions similar to PDI, but are less active. We also identified a transcript encoding a second redox inactive TRX domain b' belong to Ep72 and Ep57 subfamily. In addition, transcriptome contigs with homology to several Chaperones, including 78kDa glucose regulated protein, Hsp70, Hsp60, Hsp90, glucose regulated protein 94, different subunits of the T-complex protein 1, DNA J (Hsp40), calnexin, calreticulin, chaperonin 10kDa subunit, prefoldin superfamily and activator of Hsp90 ATPase I were also identified.

The other enzymes we have identified include a proline hydroxylase related to the enzyme involved in collagen biosynthesis. (Unrelated to the posttranslational modification of peptides, we have also identified the *egl nine *homolog-also a prolyl hydroxylase). We have identified both FK506 binding protein type peptidyl prolyl cis-trans isomerase and the cyclophilin peptidyl prolyl cis-trans isomerase. The latter type has been shown to enhance the rate of correct folding of conopeptides containing proline residues [47]. Other enzymes identified include lysyl hydroxylase, vitamin K dependent γ-glutamyl carboxylase [48,49], vitamin K epoxide reductase and peptidyl glycine alpha amidating monooxygenase.

A large number of hormones and neuro-active peptides require C-terminal amidation for full activity [50-52]; conopeptides are no exception. C-terminal amidation is a two-step process. Peptidylglycine α-hydroxylating monooxygenase (PHM) catalyzes the hydroxylation of the α-carbon of glycine and a second enzyme, peptidyl-α-hydroxy glycine α-amidating lyase (PAL) catalyzes the formation of the amidated product and glyoxylate. In *Drosophila *these two activities are carried out by separate polypeptides, whereas in other organisms (*C.elegans*, *Xenopus laevis*, human and rat) a single polypeptide carries out both activities. We discovered a single transcriptome contig encoding both PHM and PAL domains, thus C-terminal amidation of conopeptides is likely carried out by a single enzyme in *C. bullatus*.

A unique posttranslational modification first identified in *Conus *was the presence of 6-Br tryptophan in conopeptides, e.g. bromocontryphan [53], bromosleeper [54] and light sleeper [55]. Subsequently the modification was also characterized in a peptide isolated from mammalian brain [55-57]. The enzyme responsible for this modification has not been characterized. However, four different classes of haloperoxidases are known [58], which are enzymes that use heme iron/H_2_O_2_, vanadium/H_2_O_2_, FADH_2_/O_2_, and non-heme iron/O_2_/α-ketoglutarate. In the present analyses we have not identified any of the above classes of enzymes.

Another posttranslational modification is the isomerization of L-amino acids in peptides to the D-conformation [59]. The enzyme has been isolated from the funnel web spider venom [60]. At present we have not identified any transcript possibly encoding the isomerase.

### A novel method for estimating genome size

**Figure 4:**
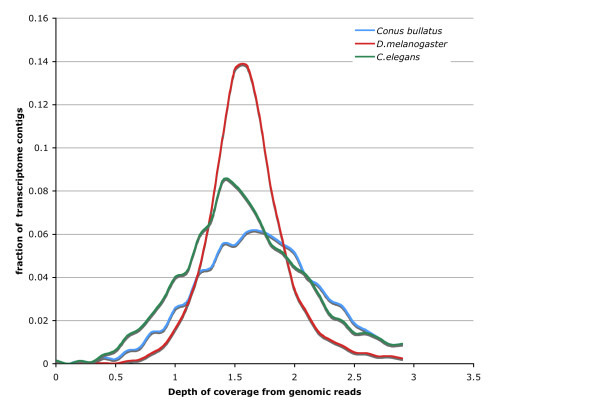
***C. bullatus *genome size estimated using Illumina reads**. Blue-line: *C. bullatus*; Red-line: *D. melanogaster*. Green line: *C. elegans*. x-axis: depth of coverage of transciptome contigs by aligned genomic reads. y-axis: frequency. In all cases the best estimate for genome size is the product of the total length of genomic reads and the mode of the frequency distribution.

We have developed a novel method for determining genome size, using 2^nd ^generation genomic and RNA-Seq reads (see Methods). For proof of principle, we first estimated the genome size of *D. melanogaster*. To do so, we simulated 4,342,253 59bp genomic reads for the fly-genome, and blasted the annotated fly transcriptome against the simulated reads (red line in Figure 4). The depth of coverage peak is at 1.50 (Figure 4). Thus, the estimated genome size for *D. melanogaster *is 4,342,253*59/1.50 = 170.8 MB. Compared to the current size of fly genome (166.6 MB), the error is 2.5%. We also estimated the genome size of *C. elegans*. This time we randomly sheared the annotated transcriptome of *C. elegans *into short contigs with the same N50 as our *C. bullatus *transcriptome assembly, and randomly selected a 57mb subset of these contigs. We did this to simulate the fragmented nature of our *de novo *transcriptome assembly. We also simulated 2,630,408 genomic *C. elegans *reads, and blasted them to the subset of simulated *C. elegans *transcriptome. As shown in Figure 4 (green line), the peak depth of coverage for the transcriptome is 1.45×. We repeated this experiment three times; there was no variance in this value. This gives us an estimate of genome size of 107.0MB, which is 6.7% higher than estimated genome size (100.3MB), again a good fit to the published genome size. For *Conus bullatus*, the estimated coverage depth is 1.70× from 4.36GB of sequence reads, thus the best estimate for the size of the *Conus bullatus *genome is 2.56 GB.

## Discussion

2^nd ^generation sequencing technologies now make it possible to probe new and emerging model organism genomes in a cost effective manner. This means that genomes and transcriptomes can be rapidly trawled for specific contents, and at the same time the organism can be evaluated for suitability of whole-genome *de novo *assembly. We have tried to accomplish both these tasks in the work reported here.

Our transcriptome analyses provide the first global view of gene expression within a *Conus *venom-duct. Several lines of evidence suggest that our dataset provides a relatively comprehensive view of this pharmacologically important tissue. First, the relative proportion of *C. bullatus *genes (as discovered by annotating out transcriptome data) assigned to different GO terms resemble those of other well annotated transcriptomes. Second, 85% of CEGMA's universally conserved eukaryotic genes are represented by one or more contigs, providing an independent estimate of the degree of completeness of the assembly. One caveat to this conclusion is that highly expressed basic house keeping genes are over represented in the CEGMA set; thus a more precise statement is that 85% of highly expressed genes are present in the RNA-seq data.

Our RNA-seq data are highly enriched for reads with conopeptide homology. The average read depth of contigs homologous to conopeptides is 102× as opposed to 33X for the remaining contigs. Interestingly, their super-family frequency spectrum roughly approximates that of the Conoserver reference collection in general [42], although some rare classes are missing.

Overall, the distribution and frequencies of GO functions, processes and locations of annotated transcriptome data closely parallel those of various carefully annotated model organism transcriptomes (Additional File 1); this fact suggests that overall, the venom-duct transcriptome is diverse, despite the highly specialized nature of this tissue. Although, as our recovery of numerous conopeptides and post-translational modification (PTM)-enzymes makes clear, its transcription is also clearly geared toward venom production. Our success at characterizing the conopeptide and candidate PTM-enzymes demonstrates the power of the RNA-Seq approach for conopeptide discovery. The conopeptide and PTM-enzymes we have discovered present new avenues for future research, as it is now possible to express these proteins in heterologous cells in order to explore interactions PTM-enzymes and their conopeptide targets [47,48,61,62].

Our genomic shotgun survey data have allowed us to characterize the *C. bullatus *genome. Our analyses indicate that it is enriched for simple repeats relative to the human genome. Characterization of its interspersed repeat populations is complicated by the lack of an adequate repeat library for RepeatMasker. To circumvent this obstacle, we developed a novel analysis method, comparing the inter-read similarity frequency spectrum of our *C. bullatus *genome reads to the inter-read similarity frequency spectrum of matched human dataset. Based upon this analysis we conclude that *C. bullatus *has higher repeat content, yet contains fewer extremely high-copy repeat species. Because this method requires no assembly or prior knowledge of a genome's repeat content, it should prove useful to others seeking to characterize the repeat contents of new and emerging model genomes.

## Conclusions

We have carried out the first transcriptome and genomic survey of a *Textilian*, *Conus bullatus*. Our RNA-seq analyses provide the first global view of transcription within a *Conus *venom duct, and demonstrate the feasibility of trawling these data for rapid discovery of new conopeptides and PTM-enzymes. We find that numerous A-superfamily peptides are expressed in the venom duct. These conopeptides are unprecedented in their structural diversity, suggesting that *Conus bullatus*, and potentially the *Textilia *clade in general, has explored novel evolutionary pathways in generating its complement of A-gene super-family peptides. Our data also provide support for the long-standing hypothesis that conopeptides are under diversifying selection. Our genomic analyses have revealed that the *C. bullatus *genome has higher content of interspersed repeats, yet fewer extremely high-copy-number repeats compared to human.

## Methods

### Preparation of RNA samples

Specimens of *Conus bullatus *were collected in the Phillippines. Each specimen was dissected to isolate the venom duct and the duct was immediately suspended in 1.0 mL RNAlater solution (Ambion, Austin, TX) at ambient temperatures, and then stored at -20 degrees Centigrade until used. Total RNA was isolated using *mir*Vana^® ^miRNA isolation kit (Ambion, Applied Biosystems CA USA) according to the manufacturer's recommendation Tissue homogenization was carried out using a tissue tearor (Model 985370, Dremel, WI, USA).

### Simulated read sets

To produce the matching sets of reads from other genomes with which to compare our *C. bullatus reads*, we randomly sampled some number of read pairs from our *Conus *dataset. Next we randomly selected substrings from an assembled target genome (e.g. human, *D. melanogaster*, etc.) having the same length and pair distances as our *Conus *reads. This matched dataset mimics the *Conus *data precisely as regards number of reads, distance between pairs, read lengths, and importantly base quality. This last feature is accomplished by mutating the simulated reads from the target genome using the base quality values of the selected *Conus *reads. These matched datasets enable many useful analyses. For example, a set of 1,000,000 randomly selected *Conus *genomic reads can be passed through RepeatMasker and the results directly compared to that produced from its matched human counterpart.

### Partial genome assembly

We generated a total of 152 million Illumina genomic reads, with read lengths of either 59bp or 60bp depnding upon run. The reads are paired-end, and have a average insertion size of 200bp. We used the 'qualityTrimmer' algorithm in the EULER-SR software package [63] to remove bad reads and trim low-quality region from reads. We then used ABySS 1.0.15 [34] for assembly, with the following parameters: c = 0, e = 2, n = 2. The k-mer size is an important factor for the quality of assembly, and in order to make an informed decision about the k-mer size, we assembled the *C. bullatus *genome with k = 25, 30, 35, 40, 45 and 50. The k-mer size of 25 generate an assembly with the best total length (201MB) and N50 (182bp). The assembly was filtered so that contigs/scaffolds with lengths less than 100 bp were removed. When aligning the genomic reads back to the *de novo *assembly, 3.6 million reads aligned.

### Assembly of the transcriptome

102 million paired-ended RNA-seq reads were generated using the Illumina sequencing platform. The read lengths for these runs were 79bp, with an average insertion size of 340bp. These reads were first filtered with EULER-SR's 'qualityTrimmer' algorithm as above, then assembled by ABySS 1.0.15 using the following parameters: c = 0, e = 2, E = 0. k-mer size of 25, 30, 35, 40, 45, 50 were tested, and the assembly at k = 35 were chosen in consideration for the total assembly size as well as N50. The assembly was filtered so that contigs/scaffolds with lengths less than 60 bp were removed.

To assess the quality of the transcriptome assembly, we aligned the RNA-seq reads back to the assembly with Bowtie. Out of 102 million reads, 31million aligned to the transcriptome under single-end alignment mode. A much smaller portion (3.2 million) of reads were aligned under paired-end mode. This is expected because our library should be enriched for short conopeptide sequences, thus many fragments should be shorter than 340bp, which will produce overlapping paired-reads that won't align under paired-end mode of Bowtie.

### Characterization of repeat content in the genomic assembly

We randomly selected 1 million Illumina reads for the genome of *Conus bullatus*. As a control, we used the reference genomes of *Aplysia californica, Caenorhabditis elegans*, *Drosophila melanogaster *and *Homo sapiens *from NCBI database. For each of the control genomes, 1 million Illumina reads with the same length and base-calling accuracy distribution were simulated. We also used a second control consisting of 100,000 real Illumina genomic reads randomly sampled from the Flatley genome [30]. We ran RepeatMasker with the '-species all' option in order to characterize all known families of interspersed repeats. These data are shown in Figure 2.

Novel repeat families with *Conus bullatus *genome were identified by running RECON over the longest genomic contigs with a total length of 30MB (masked by RepeatMasker beforehand). We then perfromed an all-by-all BLASTN of the contigs against themselves, using an E-value threshold of 1e^-8^. The blastn reports were converted into MSP files and fed to RECON to identiy genomic sequences present in no less than 10 copies in the 30MB sample sequence. 115 high-copy-number sequences were identified, and any of them that have significant homology (1e^-5^) with a UniprotKB or Repbase entry were removed from the novel interspersed repeats collection.

### Estimation of the proportion of repetitve regions

1 million genomic reads from the conus genome were randomly selected; 1 million human genomic reads were then simulated with the same length and base-calling accuracy. We aligned each set of reads to themselves with BLASTN to look for significant similarity (M = 1 N = -3 Q = 3 R = 3 W = 15 WINK = 5 filter = seg lcmask V = 1000000 B = 1000000 E = 1e-5 Z = 3000000000). The percentage of reads having each number of BLAST hit were then tallied.

To convert the number of BLAST hits to the copy-number of their corresponding genomic sequence, we simulated a genome with the same size as the human genome and the following features: 38% of this genome are comprised of unique sequence; 20% are sequences with 2 copies; 10% of the genome have 5 copies, 10 copies, 100 copies and 1000 copies each; 1% of the genome have 10,000 and 100,000 copies each. Then we simulated 1 million reads from this genome with the same length and base-calling accuracy as the Conus genomic reads and performed an all-to-all blast approach as described above. For each read generated, we tracked the copy number of the genomic region that it is extracted from. Then we calculated the average number of read partners for reads from different copy-number region. As Additional File 3 shows, the average number of read partners is correlated extremely well with the copy-number of the genomic region the read was drawn from. The equation in Additional File 3 allows us to profile the proportion of genomic regions with different copy-numbers, as shown in Figure 3.

### SNP rates

To estimate SNP rates within our transcriptome assembly, Illumina reads were aligned to contigs no shorter than 60bp in the transcriptome assembly, using Bowtie [64] with default parameters. With the samtools package, the resulting Bowtie report was converted into SAM files [65], then used to estimate the SNP ratio with samtools. We used stringent criteria to call SNPs, requiring that: 1) the SNP phred score was higher than 20; and 2) that each SNP variant was supported by at least two reads. The SNP rates within conopeptides were estimated using a same approach. We also calculated the proportion of triallelic SNPs, which is 15%, indicative of the upper bound of the false-positive rate due to mis-alignment.

### BLAST searches for conopeptides

We ran BLASTX on our transcriptomal assembly against the combined database of UniProtKB [40] and conotoxins from ConoServer [42], using the following parameters: W = 4 T = 20 filter = seg lcfilter. Contigs that hit a conopeptide as its best hit were collected as the low-stringency conopeptide set, and subsequently translated into peptides according to the reading frame identified by BLASTX. We then ran BLASTP on the low-stringency conopeptides against the combined database, using the following parameters: hitdist = 40 wordmask = seg postsw matrix = BLOSUM80. The results are filtered with E < = 3e^-5^.

### Assignment of putative conotoxins to superfamilies

We first translated each putative conotoxin conteg into peptide sequence, using the reading-frame predicted from BLASTing the RNA-seq assembly to ConoServer's collection of conopeptides. Each translated putative-conopeptide was then aligned with BLASTP to conotoxin signal peptides sequences, downloaded from ConoServer. We required all aligments to have Expect < = 1e-4, and to have at least 7 identical amino acids aligned. The best hit for each putative conopeptide is used to predict its superfamily. Overall, we were able to assign 543 putative conopeptides to a superfamily. As a control, we downloaded previously reported conopeptides from ConoServer, and randomly sheared these sequences into short oligos with the same N50 as our putative conopeptide contigs. We applied the same approach to assign these to superfamiles. Out of 3274 oligos, we were able to assign 449 to a superfamily, of which 443 (98.7%) were correct. Thus, we believe our assignment method is reasonably accurate.

### Genome size estimation

We ran WU-BLASTN over all transcriptomal contigs longer than 300bp against 73,898,732 59-mer genomic reads, with the following parameters: M = 1 N = -3 Q = 3 R = 1 wordmask seg lcmask. The coverage depth for each transcript was calculated from dividing total length of reads mapped to this transcript by its transcript length. Then the frequency distribution is shown in Figure 4. The estimated coverage depth for the genome is determined as the coverage depth with the highest frequency, which is 1.70×. The estimated genome size for *Conus bullatus *is thus 73,898,732*59/1.70 = 2.56×10^9 ^bp.

### Significance of conopeptide BLAST hits

The short reads and base quality issues combine with the short lengths of conopeptides to make identification of conopeptides in RNA-seq data difficult. Because many conopeptide transcript species are represented by only one or a few reads, the base-quality of the resulting contig is often low, especially as regards indels. All of these facts combine to make the detection of even highly conserved conopeptides problematic, because BLASTX is unable to take into account indel induced frameshifts in the contigs when calculating the significance of a hit [35], thus many real hits are not detected. Also problematic is the cysteine-rich nature of conopeptides, leading to spuriously significant hits against other non-homologous but cysteine-rich proteins, and protein domains. To control for these issues we performed a simulation to help us determine the best E-value threshold for a conopeptide hits in RNA-seq data. We first ran WU-BLASTP [36] on our transcriptome assembly against the combined database of UniProtKB [40] and conopeptides from ConoServer [42]. In total, 6,677 peptides were found to have a known conopeptide as its best hit. We then plotted the E-value distribution of the BLAST results for the best HSPs (Additional File 4). Next, we randomly permuted the sequences of each of our 6,677 *C. bullatus *contigs with conopeptide hits using a Fisher-Yates shuffle [66]. We then ran BLASTP using the permuted peptides against the combined UniProtKB and conotoxins database, and plotted the E-value distribution for all hits. Presumably, the latter plot should represent the background distribution of insignificant BLAST hits. We found that only 5% of the hits in the permuted peptide set have an E-value of lower than 3e-5, while in the putative conopeptide set, the percentage is 48%. Thus we used E < = 3e^-5 ^as the E-value threshold for our BLASTP searches for conopeptides.

### Data and software availability

The read-simulation tool and data (transcriptome assembly, genomic assembly, putative conotoxin sequences and post-translational modification enzymes) can be downloaded at http://derringer.genetics.utah.edu/conus/. The software is open source.

## Authors' contributions

HH, PB and MY wrote the paper. HH wrote software and carried out experiments. PB annotated and analyzed results. MY, PB and BO conceived of the project and oversaw the experiments. All Authors read and approved the final manuscript.

## Supplementary Material

Additional File 1 **GO analyses**. GO term abundance for molecular function. In each organism (colored as in the legend), each transcript was assigned applicable high-level generic GO slim terms. The occurrence of each GO term was counted and converted into frequency among all GO terms. Similar congruency between transcriptomes was seen for GO process and location terms. Click here for file

Additional File 2 **Proteins involved in post-translational modification**. Annotated list of proteins that are presumed to participate in conotoxin synthesis and posttranslational modification. Deduced from conceptual translation of transcripts (ESTs) present in the venom duct. Click here for file

Additional File 3 **Correlation between Average read partner number (from all-by-all BLAST) and actual copy number of corresponding genomic sequence**. A human-size genome is simulated so that certain fractions of the sequence are present in 1 copy, 2 copies, 5 copies, 10 copies, 100 copies, 1000 copies, 10,000 copies and 100,000 copies. The average read partner count for reads simulated from each group is calculated and used for the plot. Click here for file

Additional File 4 **Determining the appropriate BLAST E-value for identification of conotopeptides**. Red-line: E-value frequencies for all contigs with conopeptide homology. Blue-line:E-value frequencies for the same set of contigs after permutation. X-axis: frequency; y-axis E-value. 5% of the permuted contigs have an E-value of less than 3e-5, compared to 45% of the native set. Thus, we choose 3e-5 as our cutoff threshold for a 0.05 confidence level. Click here for file
