# Supplementary figures and images for: Characterization of the Conus bullatus genome and its venom-duct transcriptome
Source: BMC Genomics. 2011 Jan 25;12:60. doi: 10.1186/1471-2164-12-60 (PMC3040727; doi:10.1186/1471-2164-12-60)

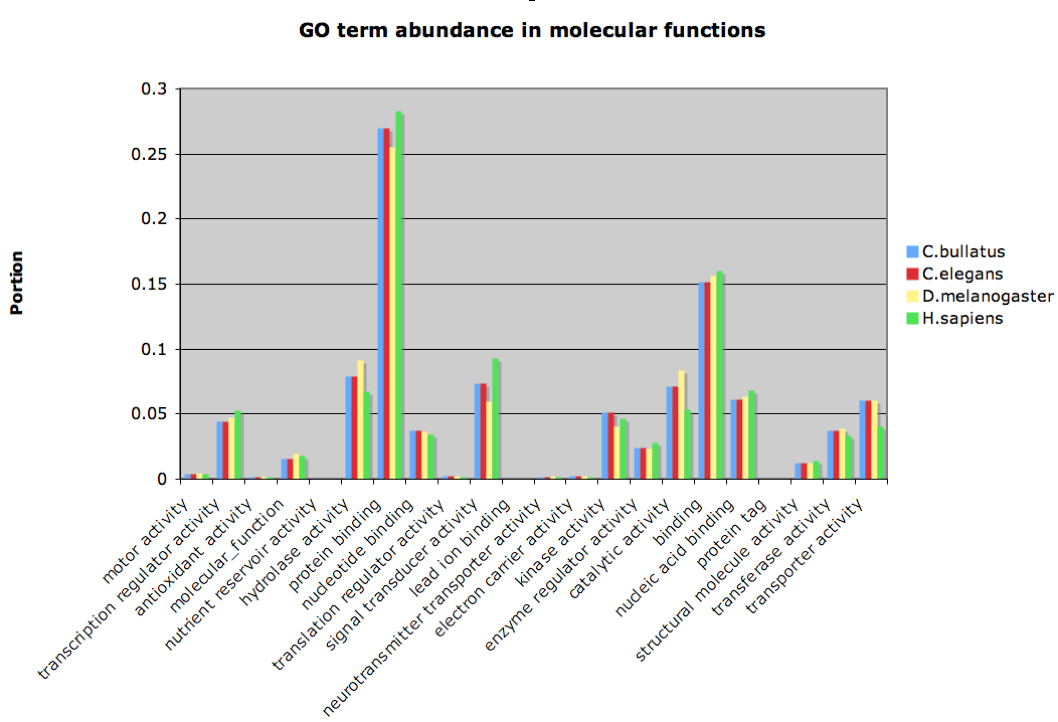

Supplement: Additional File 1 — GO analyses. GO term abundance for molecular function. In each organism (colored as in the legend), each transcript was assigned applicable high-level generic GO slim terms. The occurrence of each GO term was counted and converted into frequency among all GO terms. Similar congruency between transcriptomes was seen for GO process and location terms. [file 1471-2164-12-60-S1.TIFF]

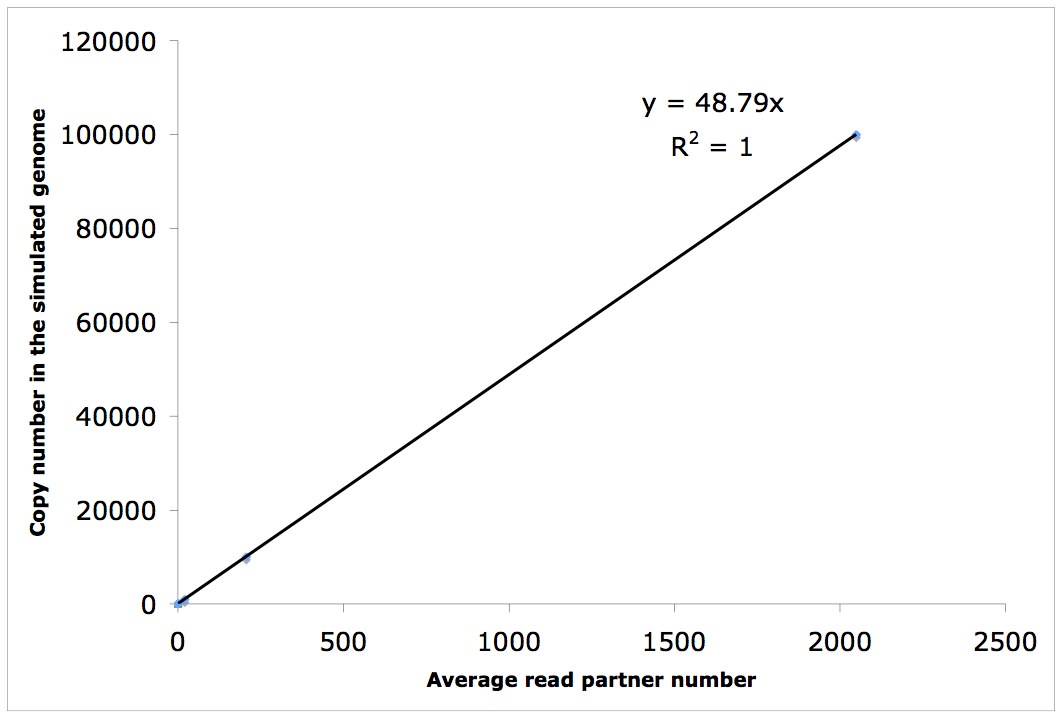

Supplement: Additional File 3 — Correlation between Average read partner number (from all-by-all BLAST) and actual copy number of corresponding genomic sequence. A human-size genome is simulated so that certain fractions of the sequence are present in 1 copy, 2 copies, 5 copies, 10 copies, 100 copies, 1000 copies, 10,000 copies and 100,000 copies. The average read partner count for reads simulated from each group is calculated and used for the plot. [file 1471-2164-12-60-S3.TIFF]

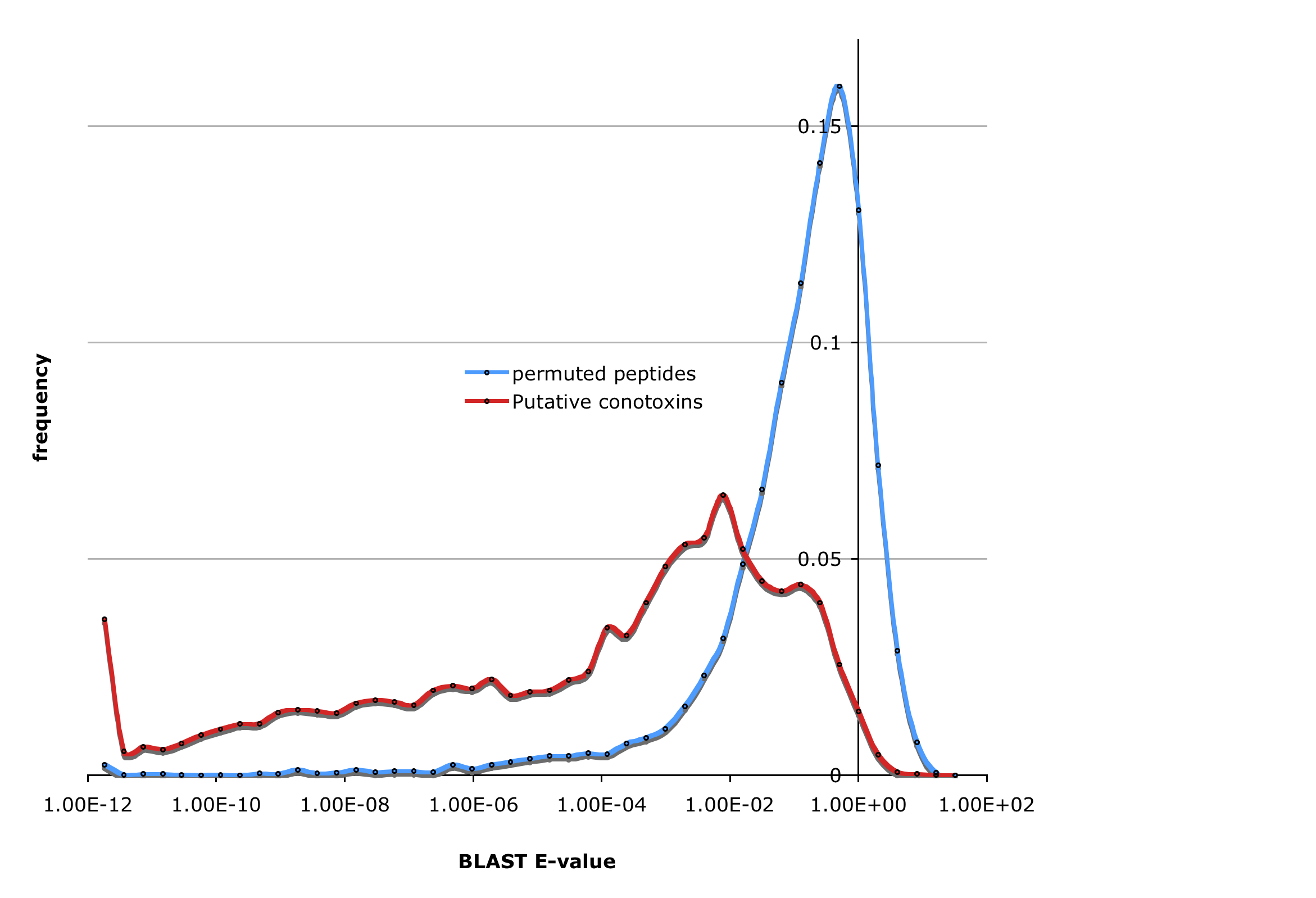

Supplement: Additional File 4 — Determining the appropriate BLAST E-value for identification of conotopeptides. Red-line: E-value frequencies for all contigs with conopeptide homology. Blue-line:E-value frequencies for the same set of contigs after permutation. X-axis: frequency; y-axis E-value. 5% of the permuted contigs have an E-value of less than 3e-5, compared to 45% of the native set. Thus, we choose 3e-5 as our cutoff threshold for a 0.05 confidence level. [file 1471-2164-12-60-S4.TIFF]
